# The spatial buildup of nonlinear compression in the cochlea

**DOI:** 10.3389/fncel.2024.1450115

**Published:** 2025-01-29

**Authors:** Kostas Kondylidis, Anna Vavakou, Marcel van der Heijden

**Affiliations:** ^1^Dept. Neuroscience, Erasmus MC, Rotterdam, Netherlands; ^2^GERS-GeoEND, IFSTTAR, Université Gustave Eiffel, Nantes, France; ^3^Institute for Auditory Neuroscience, University Medical Center, Göttingen, Germany

**Keywords:** cochlear mechanics, basilar membrane, compression, traveling wave, distortion products

## Abstract

In the mammalian cochlea, the transduction from vibrations to inner hair cell receptor currents is preceded by a stage of mechanical pre-processing that involves a rapid, strongly nonlinear compression. The mechanisms by which the cochlea realizes this dynamic compression are still poorly understood. Previous work by our group suggested that compression does not occur locally, but is realized by a cascade of weakly nonlinear elements along the cochlear partition. The resulting progressive accumulation of nonlinearity was termed the spatial buildup of compression. Here we studied mechanical compression in the basal turn of the sensitive gerbil cochlea using optical coherence tomography. We recorded vibrations at multiple positions along the length of the cochlear partition. Such longitudinal studies were virtually impossible with previous techniques. Using a tailored two-tone stimulus we quantified the spatial profile of compression. We found that the amount of compression grew gradually in an intensity-dependent fashion along our measurement stretch, as we moved apically toward the place of maximum vibration. This gradual buildup of compression was not mirrored by a gradual reduction beyond the peak. In fact the amount of compression accumulated even beyond the peak. This asymmetric pattern supports the view that mechanical compression is realized in a cascaded, distributed fashion which hinges on the traveling wave nature of cochlear vibrations.

## 1 Introduction

In the mammalian ear, the huge (>100-dB) dynamic range of audible sounds is compressed to less than 40 dB to fit the much smaller dynamic range of inner hair cell transduction. The exact mechanisms underlying this mechanical sensitivity control are unknown, but outer hair cells play a crucial role; their loss causes a linearization of the cochlear response (Patuzzi et al., 1989; Ruggero and Rich, 1991).

Experimentally, nonlinear compression in the cochlea has been quantified by the slopes of input–output functions of basilar membrane vibrations. In a sensitive cochlea, the slopes observed at a single position can vary from linearity (1 dB/dB) well below the best frequency to values as low as 0.1 dB/dB above best frequency (e.g., Rhode, 2007). The strong frequency dependence also shows up in the nonlinear interaction between different frequency components. The response to one tone (probe) is suppressed by the introduction of another tone (suppressor), On the basilar membrane (BM), the amount of suppression depends in a nontrivial way on the frequencies of both the probe and the suppressor (e.g., Cooper, 1996).

Mechanical compression and its strong frequency dependence have a prominent effect on subsequent stages of the auditory pathway. Auditory nerve responses to single tones show frequency dependent growth rates consistent with single-tone BM responses (Cooper and Yates, 1994), and two-tone suppression in auditory nerve responses (Abbas and Sachs, 1976; Delgutte, 1990) is generally consistent with two-tone suppression on the BM, including its complex frequency dependence. The classical psychoacoustic study of Wegel and Lane (1924) reported how the audibility of one tone (signal) is affected by the introduction of a second tone (masker). The frequency dependence of these masking data and later refinements (e.g., Zwicker and Jaroszewski, 1982) is again consistent with the suppression data on the BM, suggesting a strong role of cochlear nonlinearity in auditory off-frequency masking.

Most of the BM data described above were obtained in the base of the cochlea, but the corresponding effects in the auditory nerve data and psychoacoustics span a large range of frequencies. The entanglement of compression and frequency analysis thus appears to be a universal feature of mammalian hearing, and it is desirable to try and capture the complexity in terms of a limited number of unifying principles. This may be a first step toward understanding the physiological mechanisms. To be sure, several detailed cochlear models reproduce the basic frequency dependence of compression and suppression; early examples include Lyon (1982), Kanis and de Boer (1994), and Geisler and Sang (1995), but these models differ among them in many basic aspects. In our view it is helpful to transcend the realm of detailed models and rather try to formulate an overarching framework to explain the universal features of cochlear compression and suppression.

The basic features of compression are as follows (see also Robles and Ruggero, 2001; Cooper, 2004). At a given cochlear location the amount of compression, quantified by the slopes of input-out functions, depends strongly on stimulus frequency. The frequency dependent growth is observed not only when single tones are presented, but also when multiple frequencies are presented simultaneously (Versteegh and Van der Heijden, 2012). When viewed in the frequency domain, the entanglement is reflected by a strong intensity dependence of frequency selectivity (Rhode, 2007). Cochlear nonlinearity has been most extensively studied from recordings of basilar membrane (BM) vibrations in the basal turns, but more recently a similar frequency dependence of compression was reported in BM vibration in the second turn of the gerbil cochlea (2.5-kHz region; Meenderink et al., 2022). The main characteristics of cochlear compression at the level of the BM can be summarized as follows.

- Cochlear compression is very fast but not instantaneous (Cooper and Van der Heijden, 2016).
- It approximates a multiband gain control, meaning that sensitivity is more or less independently regulated in different frequency bands (Cooper, 2004; Robles and Ruggero, 2001).
- At high intensities the independence of frequency bands breaks down asymmetrically. Low-frequency components start to suppress the sensitivity to sounds at much higher frequencies (upward spread of masking; Wegel and Lane, 1924).
- At a given BM location the degree of compression, expressed as the slope in dB/dB of I/O functions, is strongly frequency dependent. Components more than ~1/2 octave below the best frequency show linear growth (1 dB/dB). With increasing frequency the slopes become increasingly more compressive (<1 dB/dB), and this trend continues beyond the best frequency (Rhode, 2007; Cooper et al., 2018).

Thus the nonlinear character of BM responses is largely restricted to a narrow band of frequencies around the best frequency, often called the “tip” of the tuning curve. When studying cochlear structures other than the BM, additional complexities are encountered. Superimposed onto the type of narrowband compression observed in the BM, mechanical responses inside the organ of Corti also show a wideband compression (Rhode and Cooper, 1996; Cooper and Dong, 2003; Gao et al., 2014; Cooper et al., 2018) that seems to have a different origin and is physiologically more robust (Strimbu et al., 2020). In the present study this wideband nonlinearity is only briefly touched upon and we will mainly restrict the analysis to the “classical,” narrowly tuned, compression of BM vibrations.

A previous experimental study from our lab (Versteegh and Van der Heijden, 2013) analyzed the entanglement of frequency selectivity and compression by assessing the mutual suppression of different frequency components. The independent variation of the suppressor frequency and probe frequency resulted in a rich data set that was not easy to summarize. A large number of systematic effects were observed which at first glance lacked an obvious relationship. Further analysis, however, revealed that a marked simplification and unification of the bewildering set of observations could be achieved when invoking arguments based on cochlear tonotopy, i.e., the systematic dependence of frequency tuning on cochlear location. By invoking tonotopy, the frequency dependent effects could be interpreted in terms of the *spatial* distribution of nonlinear effects along the length of the BM. The analysis underscored the essential role of traveling waves in the explanation of the complexities of cochlear compression, an ingredient that had rarely been systematically discussed before, with the exception of a very early nonlinear model by Kim et al. (1973). Versteegh and Van der Heijden (2013) argued that “many seemingly unrelated aspects of compression and suppression are in fact consequences of the gradual accumulation of nonlinearity along the travel direction. Likewise, the complex phase effects were found to reflect systematic changes in the local propagation speed of traveling waves.” They called this the *spatial buildup* of cochlear compression. A more recent analysis by Altoè et al. (2021) elaborates on these ideas.

The key feature of this conceptual framework is the concept of forward gain. At each point along the traveling wave, the gain with which the vibrations are conveyed to the next segment is variable, and depends on the local vibration amplitude. This is a form of negative feedback: larger local vibrations cause a reduction in the local forward gain and vice versa. From a functional perspective, the local character of the feedback allows it to be very fast—in principle as fast as the ability of single outer hair cells to convert their sensory input to a mechanical response. At the same time, the cascaded configuration allows the nonlinear contribution of each single outer hair cell to be very modest. Instead of burdening a single local structure with realizing an enormous (>50 dB) variation in sensitivity, a cascaded configuration distributes the work among a large number of hair cells, each of which need to adjust its gain by a small faction. For instance, a chain of 100 elements, each of which varies its gain by just 0.5 dB, results in a total gain variation of 50 dB. A cascaded configuration is in marked contrast to a set of discrete nonlinear oscillators (e.g., Jülicher et al., 2001), each of which would have to realize the large amount of gain variation observed in the sensitive cochlea.

**Figure 1:**
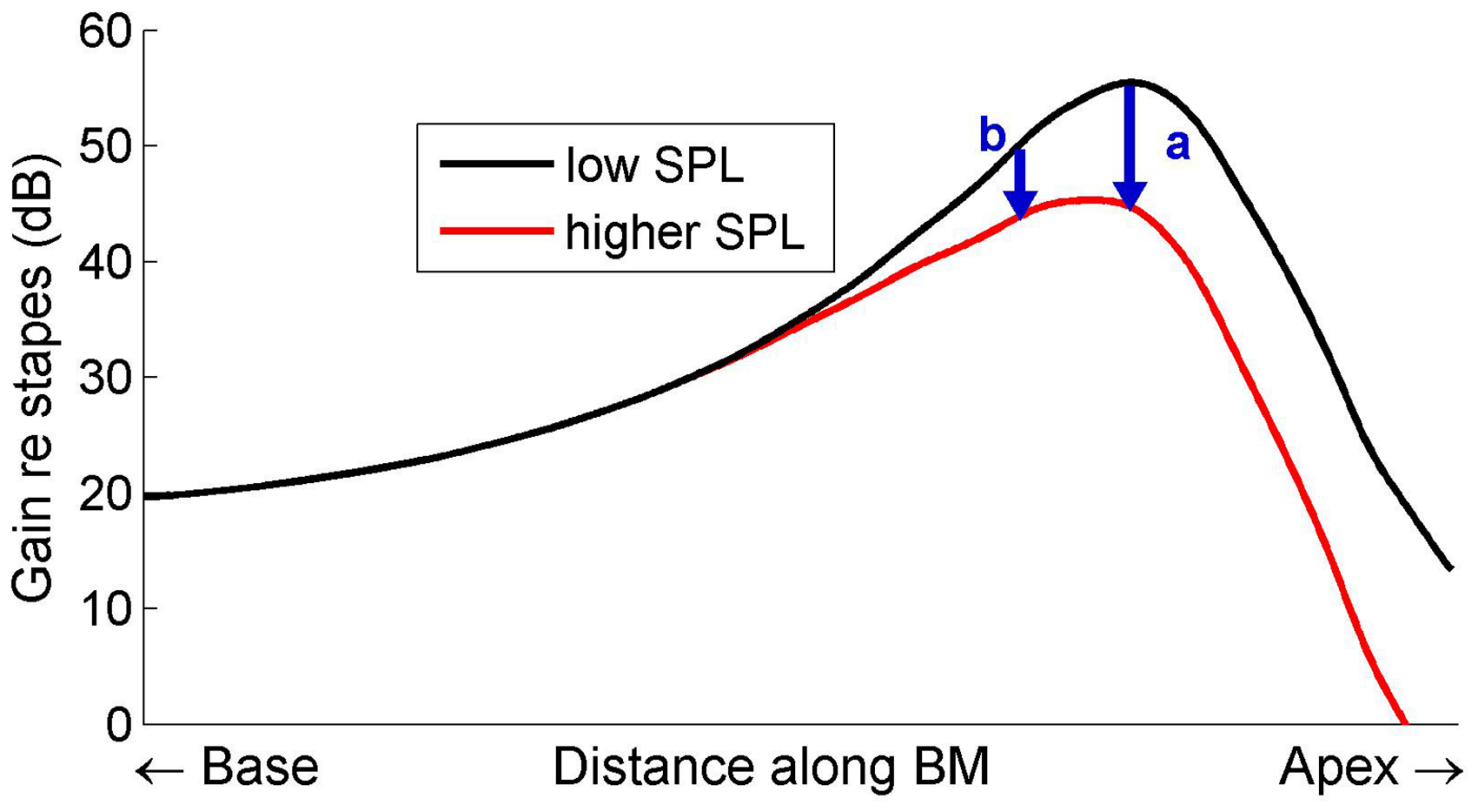
Schematic diagram illustrating how compressive growth of BM vibrations depends on longitudinal location. The curves represent the displacement patterns on the BM, normalized to middle-ear motion, evoked by a single tone presented at two intensities. The upper curve represents a low-intensity, linear, response. Increasing the intensity decreases the local forward gain of the traveling wave (e.g., by increased damping), resulting in a progressively smaller gain (lower curve). The arrows mark the amount of compression at two positions (b basal to a). The gradual divergence of the two curves causes the amount of compression to increase from base to apex.

One of the complexities of cochlear compression that finds a natural explanation in the conceptual framework of spatial buildup is the frequency dependence of the growth of compression. This is illustrated in Figure 1 by comparing the normalized spatial vibration patterns for different sound intensities.

If compression is indeed achieved by a regulation of local forward gain (e.g., through variable damping) the amount of compression for a single tone is seen to gradually accumulate from base to apex. A simple “scaling” (tonotopy) argument then concludes the explanation of the frequency dependence of the slopes of I/O function at single BM positions. A key aspect is the continued accumulation of compression beyond the peak.

The experimental data that underpinned the spatial buildup of compression in the study of Versteegh and Van der Heijden (2013) existed of single-point vibration recordings on the BM. The spatial aspect was analyzed indirectly, by invoking “scaling.” Specifically, uniform changes in stimulus frequency were used as a proxy for changing the longitudinal recording position. Likewise, recent experimental studies designed to test the spatial buildup of cochlear nonlinearity (Charaziak et al., 2020; Altoè and Charaziak, 2023) used “scaling” to extrapolate single-point BM data to spatial excitation patterns. The use of scaling is understandably popular among modelers, but its experimental basis is feeble. Neither its quantitative accuracy nor the scope of its validity has been subjected to dedicated tests. For this reason, Versteegh and Van der Heijden (2013) stressed the need for multipoint data to explore the spatial buildup of cochlear compression more directly. The present study presents such a spatial test.

We studied mechanical compression in the basal turn of the sensitive gerbil cochlea. The novelty in this work lies in the recording of vibration patterns at multiple longitudinal positions along the cochlear partition. The use of optical coherence vibrometry (OCT) for our experiments made these longitudinal recording series relatively straightforward; such spatial studies were extremely difficult with previous techniques (Ren, 2002). Using a tailored two-tone stimulus we accurately quantified the spatial profile of compression. We found the amount of compression to grow gradually in an intensity-dependent fashion along our measurement stretch, as we move apically toward the place of maximum vibration. Our results confirm and quantify the spatial buildup of compression, suggesting that outer hair cells realize mechanical compression in a cascaded, distributed fashion. This process of gradual accumulation critically depends on the traveling wave nature of cochlear vibrations.

A preliminary version of this work was presented at the 14th International Mechanics of Hearing Workshop in Helsingør, Denmark, July 2022.

## 2 Methods

### 2.1 General idea

Effective I/O functions were obtained by presenting a pair of equal-amplitude tones differing in frequency by 20 Hz. The resulting 20-Hz “beating” pattern affords a quasistatic variation of the sound pressure level (SPL). This dynamic method of obtaining I/O functions is efficient and robust, and 20 Hz is slow enough for the BM response to be free of hysteresis (Cooper and Van der Heijden, 2016). By recording BM responses along a longitudinal stretch (up to 700 μm long), we assessed how the nonlinear compression developed along the propagation direction of the traveling wave.

### 2.2 Animal preparation

Experiments were performed in accordance with the guidelines of the Animal Care and Use Committee at Erasmus MC. Sound evoked vibrations were recorded from the stapes and cochlear partition of healthy young gerbils (*n* = 18, aged 49–190 days, weight range 57–88 g). This includes experiments used for optimizing the stimulus and recording protocols. Animals were anesthetized using intraperitoneal injections of ketamine (80 mg/kg) and xylazine (12 mg/kg), with no recovery allowed at the end of the experiments. Maintenance (¼) doses of the anesthetic were given at intervals of between 10 and 60 min, as required to abolish pedal withdrawal reflexes. Animals were tracheotomized, but self-ventilating. Core temperatures were maintained at 38 degrees Celsius using a thermostatically controlled heating pad. The pinna and external meatus of the left ear was retracted and a 4×6 mm wide opening was made into the postero-lateral bulla to expose the basal aspects of the cochlea, including the stapes and the round window. A paper wick was used to prevent any buildup of fluid in the round window recess.

Experiments took place in a sound-proof chamber, with the animals supported in a goniometric cradle mounted on a vibration isolated table. Imaging and vibration measurements were made in the first turn of the cochlea, as viewed through the intact round window membrane. All measurements were made under open-bulla conditions, but the cochlea itself was intact.

### 2.3 OCT vibrometry

**Figure 2:**
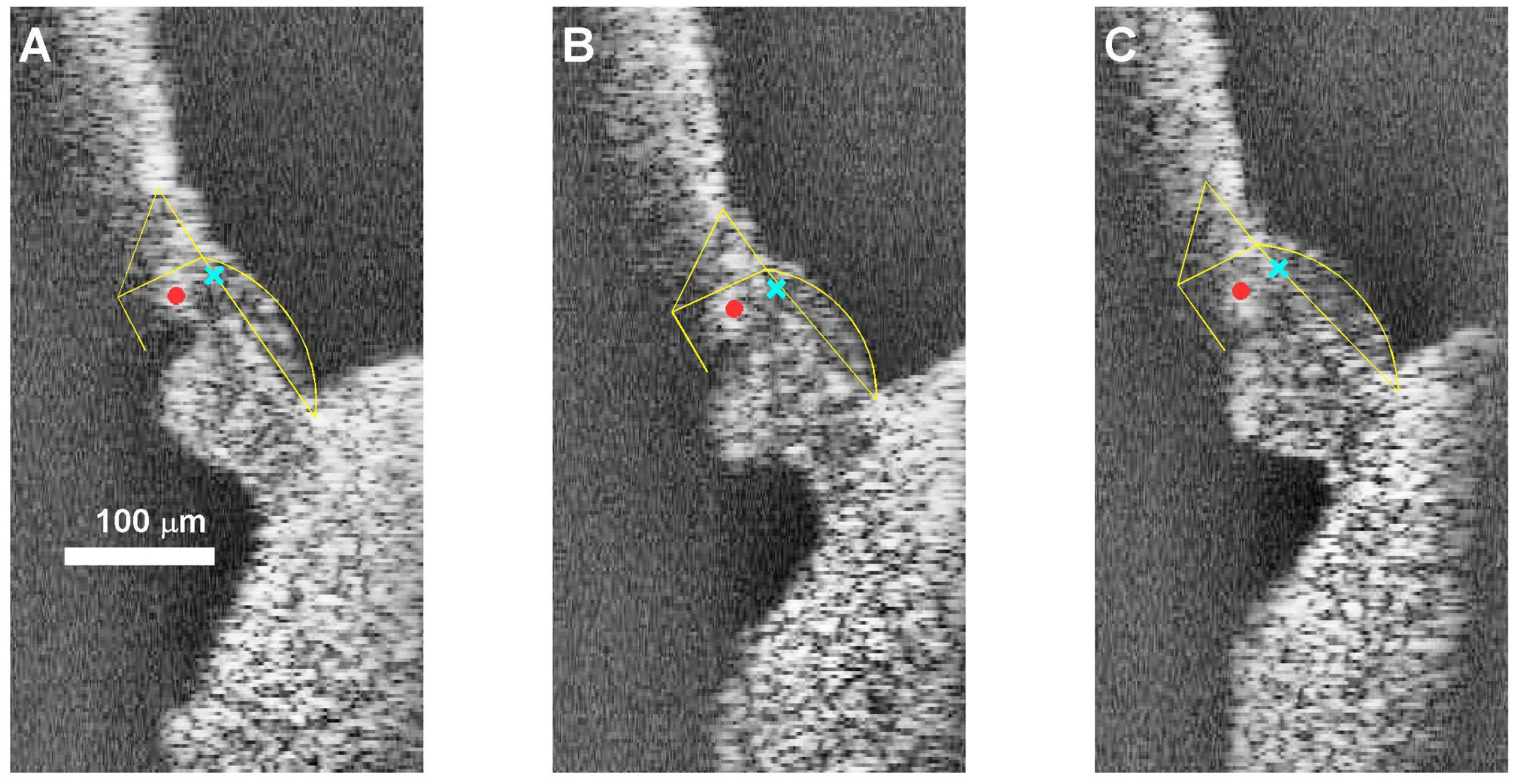
B-scans illustrating the targets of the vibration recordings in this study. **(A)** Most basal position in cochlea RG211043. **(B)** Near-middle (#6/10) position **(C)** most apical position. In all three panels, Yellow frames mark the location of the two zones of the basilar membrane, the pillar cells and the reticular lamina. The cyan X marks the recording position for basilar membrane recording, which constitute the majority of our data (Figures 4–8). The red dot shows the “hotspot” location (see text) that was used for the data in Figure 9. The scale bar applies to all three panels.

For a detailed description of the OCT recording system (see Cooper et al., 2018). An externally-triggered spectral domain optical coherence tomography system (ThorLabs Telesto III; central wavelength, 1,300 nm) was used for imaging and vibrometry. The system provided cross-sectional (B-scan) and axial images (A- scans and M-scans) phase-locked to an acoustic stimulation system (Tucker Davies Technologies system III) with a sampling rate of 111.6 kHz. Parallel series of intra-cochlear images (B-scans) were used to identify the anatomical structures of interest and to aim the beam for the recording of time series (M-scan) during acoustic stimulation. For the majority of recordings reported here, we aimed at the portion of the BM near the feet of the Deiters’ cells, which is the point of largest motion of the BM (Cooper and Van der Heijden, 2018). An exception are the recordings shown in Figure 9, which were obtained from the junction of Deiters’ cells and outer hair cells, near the center of the “hotspot” in Cooper et al. (2018). Figure 2 illustrates the targets of the vibration recordings.

For each animal, we recorded motion responses along one longitudinal stretch on the cochlea. The length of longitudinal stretches varied across experiments (290–695 μm), as the viewing angle through the round window was not identical. Consequently, the distance between recording positions (60–100 μm; 10 positions per cochlear stretch) also varied. Having the anatomical information from the B-scan images (radial and depth direction), and the distance between adjacent B-scans (longitudinal direction), we determined the Euclidian distance between neighboring measurement positions. Acoustic stimuli were subsequently presented, and vibration measurements (M-scans) were obtained for each position.

The acquisition of complete longitudinal series in a single cochlea required near-perfect mechanical and physiological stability. Buildup of fluid on the round window membrane, for instance, changes the optical path of the recording beam and disrupts the measurement series. Such artifacts were identified based on the comparison of pre- and pos-recording B-scans. Any deterioration in sensitivity was tested by a similar comparison of responses to low-SPL tone complexes. Data from 4 animals are shown in this study; they were selected for mechanical and physiological stability during complete recording series.

### 2.4 Acoustic stimulus and analysis of the vibrations

Stimuli generated by a personal computer with custom MATLAB-software were fed through a 24-bit D/A-channel (RX6; Tucker-Davis Technologies (TDT)) at 111.6 kHz. A programmable attenuator (PA5; TDT) followed by an amplifier (SA1; TDT) conditioned the signal before a speaker (CF1; TDT) played the stimuli. The speaker was connected to a sound delivery probe sealed to the ear canal with Vaseline. The sound system varied less than 4 dB in the 5–25 kHz range after correcting for the acoustic transfer of the probe.

To determine the best frequency of the recording locations, we used 41-component irregularly spaced (Zwuis) tone complexes presented at 20–70 dB SPL per tone. The best frequency of each recording position was determined by peak fitting of the magnitude-versus-frequency curve at the lowest SPL at which the data permitted such curve fitting (typically, 30 dB SPL). For the main recordings of the experiment we used 11.5-s long, equal amplitude tone pairs presented at 70 dB SPL per tone. The two simultaneous tones were spaced 20 Hz apart. The center frequency was typically chosen to match the best frequency of the most apical BM location within the longitudinal stretch available. The same stimulus was repeated while visiting each of the locations along the longitudinal stretch. If time permitted, the center frequency was varied in 1-kHz steps, repeating the longitudinal recording series for each frequency.

**Figure 3:**
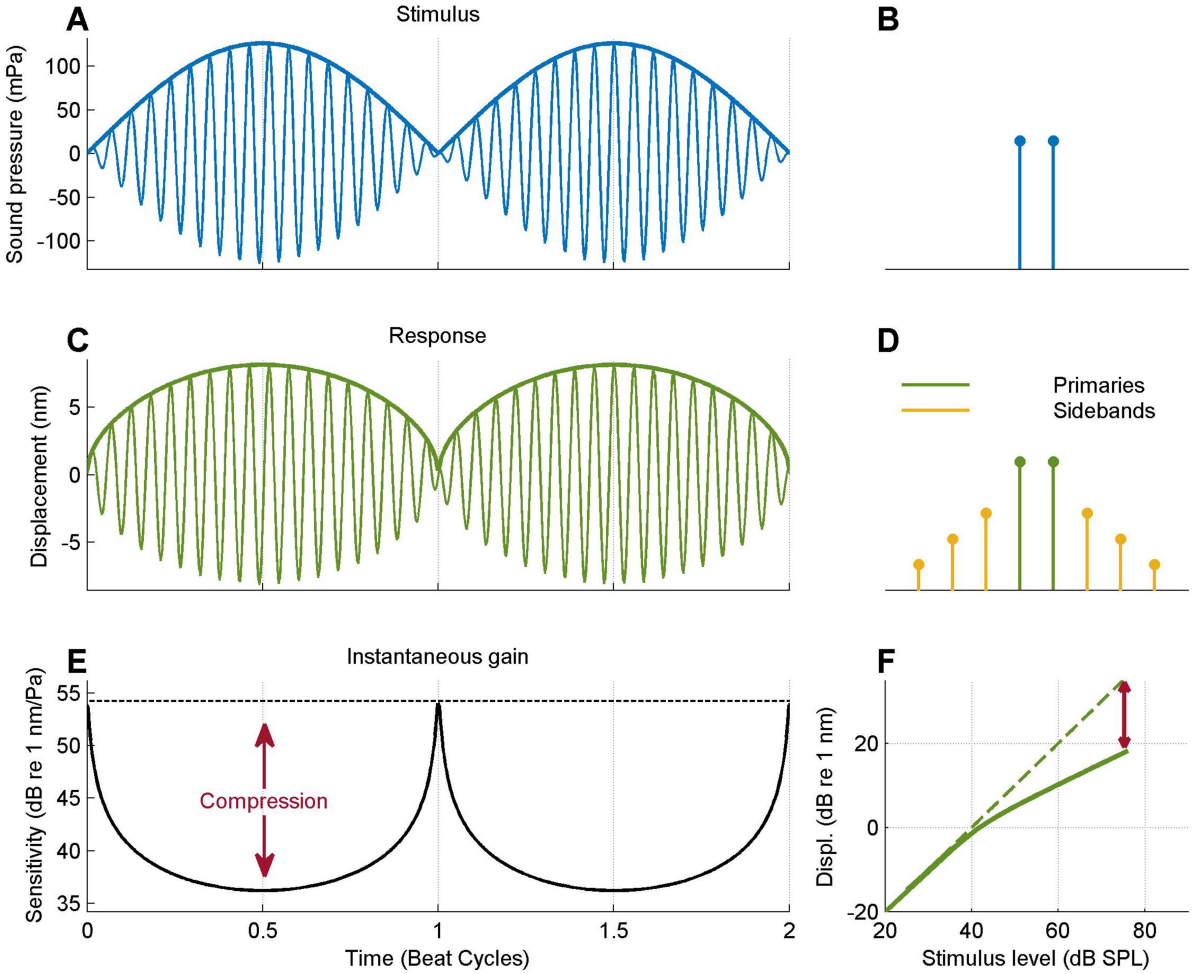
Data analysis illustrated using a model response. **(A)** Two-tone stimulus waveform (thin line) and envelope (thick line). The equal amplitude tones cause a periodic perfect cancelation. **(B)** Frequency spectrum of the stimulus, showing the two equal-amplitude primaries. **(C)** Simulated response waveform obtained by numerically compressing the waveform (0.5 dB/dB; see text) and response envelope (thick line). Note the flat-topped envelope compared to the stimulus. **(D)** Spectrum of the compressed waveform, consisting of the primary tones plus odd-order intermodulation products (“sidebands”). **(E)** Instantaneous gain or sensitivity, obtained by computing the ratio of response envelope and stimulus envelope. Red arrow, amount of instantaneous compression. **(F)** I/O curve obtained by plotting the response envelope against the stimulus envelope. Dashed line shows the extrapolation of the low-SPL linear I/O to higher SPLS; red arrow shows the amount of compression at the highest instantaneous SPL.

The basic analysis of the OCT vibrometry data is described in our previous work (Cooper et al., 2018). The cochlear vibrations in response to the tone pairs were first analyzed in the spectral domain: response components at the primary frequencies *f*_1_ and *f*_2_ of the stimulus as well as the odd intermodulation components at frequencies *f*_1_ ± *n* (*f*_2_-*f*_1_), with *n* = 1,2,…, were subjected to a Rayleigh test for significance of phase locking to the stimulus (Versteegh and Van der Heijden, 2012). Only components with a confidence level *p* < 0.001 were admitted to the analysis. From the multi-tone response thus obtained, further analysis was performed in both the spectral and temporal domains as described in the Results section (see Figure 3).

### 2.5 Numerical simulation of compression

For the illustration of the data analysis in Results (Figure 3) we used a numerical simulation of a compressed two-tone response *y*(*t*), equal to


(1) $$y\left(t\right)=\frac{x\left(t\right)}{\sqrt[{4}]{E^{2}\left(t\right)+E_{0}^{2}}}\text{,}$$

where *x*(*t*) is the two-tone stimulus


(2) $$x\left(t\right)=\cos\omega_{1}t+\cos\omega_{2}t\text{,}$$

*E*(*t*) is the envelope of the stimulus:


(3) $$E\left(t\right)=2|\cos\frac{1}{2}\left(\omega_{2}-\omega_{1}\right)t|$$

and *E*_0_ is a constant. This nonlinear map realizes an instantaneous power-law compression of 0.5 dB/dB, with a linear behavior at small (<<*E*_0_) amplitudes (Van der Heijden, 2005).

## 3 Results

### 3.1 Analysis of a numerically compressed waveform

In order to explain the analysis of the two-tone responses, we illustrate the analysis steps using artificial data obtained by subjecting an equal-amplitude tone pair to an instantaneous power-law compression with linear behavior at small amplitudes (see Methods, 2.4).

### 3.2 Buildup of compression toward the traveling wave peak

The two-tone stimulus (Figure 3A) shows a 20-Hz, beating pattern with sharp minima due to perfect periodic cancelation. The stimulus envelope is sinusoidal. The stimulus spectrum (Figure 3B)consists of the two stimulus components. The compressive transformation (see Methods, Equation 1) results in a response waveform (Figure 3C) with flattened envelope peaks; the envelope minima are undistorted as the transformation is linear at low amplitudes. The nonlinear transformation creates intermodulation products that occur as sidebands flanking the primaries in the response spectrum (Figure 3D). The instantaneous gain, expressed as the ratio of response envelope and stimulus envelope various in a periodic fashion (Figure 3E). The gain peaks at the stimulus minima and is maximally reduced at the envelope maxima. Instantaneous compression is defined as the difference between the time-varying gain and its peak value (red arrows in Figure 3E). I/O curves are obtained by plotting response envelope directly against stimulus envelope (Figure 3F) on a double logarithmic scale. This brings out the linear growth at low SPLs and the compressive (here, 0.5-dB/dB) growth at higher SPLs. Compression is indicated in Figure 3E (red arrow) as the difference in dB between the actual response magnitude and the value derived from linear extrapolation of the low-SPL I/O curve (dashed line in Figure 3E).

**Figure 4:**
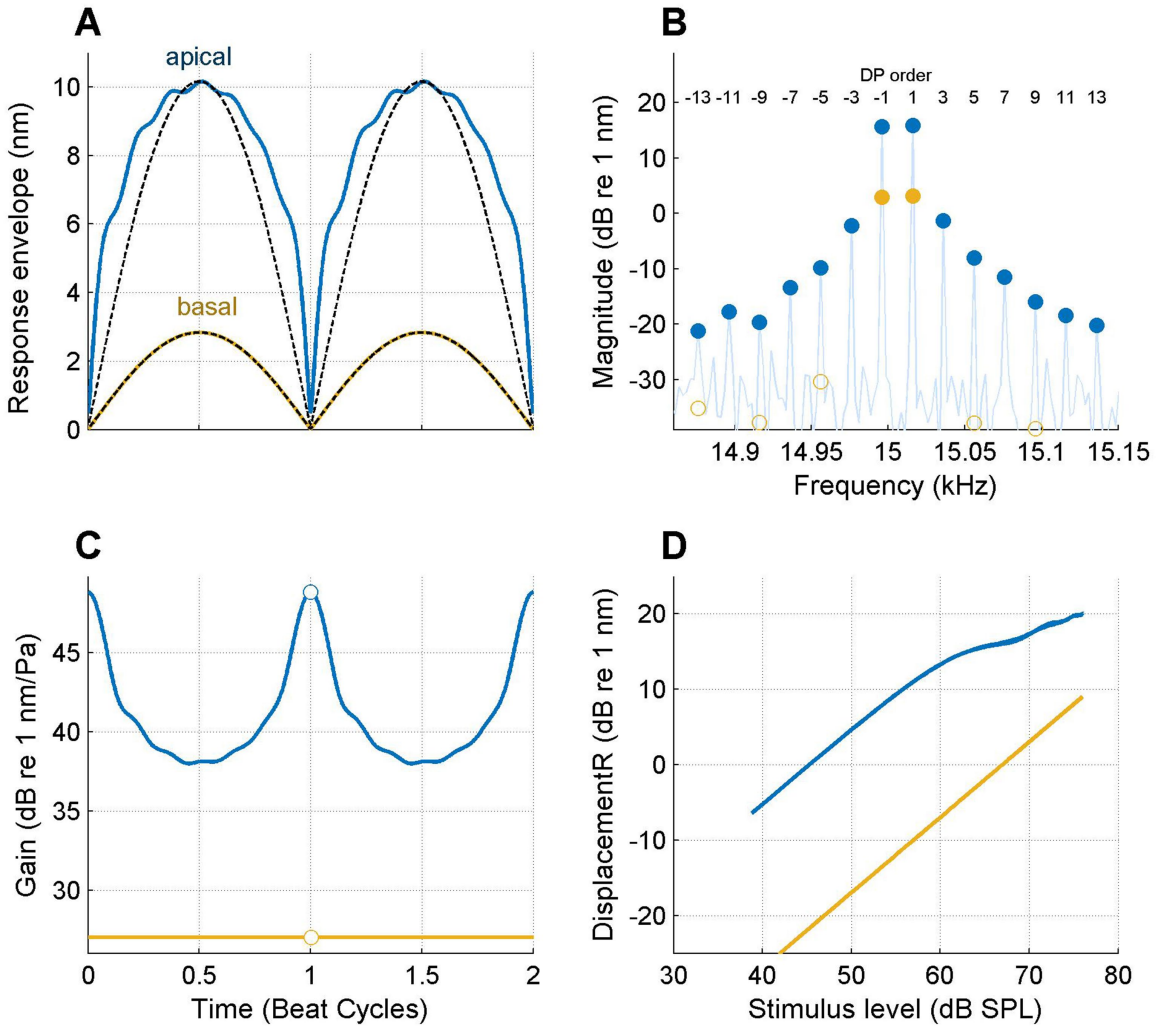
Longitudinal variation of nonlinear compression on the BM. Responses to the same 15-kHz tone pair are shown for two locations separated by 601 μm. **(A)** Response envelopes at the two locations as indicated in the graph. Black dashed lines: sinusoidal shapes for comparison. **(B)** Response spectrum. Filled symbols are stimulus or DP components that passed the *p* = 0.001 Rayleigh tests for significant phase locking to the stimulus (see text). Open symbols indicate components at the same frequencies that failed the test. **(C)** Instantaneous gain. **(D)** I/O curves. Best frequencies: 21.6 kHz (basal; ochre lines and symbols) and 15.4 kHz (apical; blue lines and symbols). Experiment RG211043.

Figure 4 shows the analysis of vibrations recorded on the BM in the basal turn of a sensitive gerbil cochlea in response to a tone pair centered at 15 kHz. Each panel compares the responses to the same stimulus recorded at two longitudinal locations of the same cochlea separated by 601 μm. The response at the basal location (ochre curves in Figure 4) is linear, as reflected by the sinusoidal response envelope (Figure 4A), the absence of sidebands (Figure 4B), the constant gain (Figure 4C) and the linear, 1-dB/dB slope of the I/O curve (Figure 4D). In marked contrast, the recordings from the apical location show a flattened envelope (blue curves in Figure 4A); a large family of distortion products of order 3–13 (Figure 4B), a time-varying gain with a range of ~10 dB (Figure 4C) and an I/O function with a clear compressive segment at the higher SPLs (Figure 4D).

**Figure 5:**
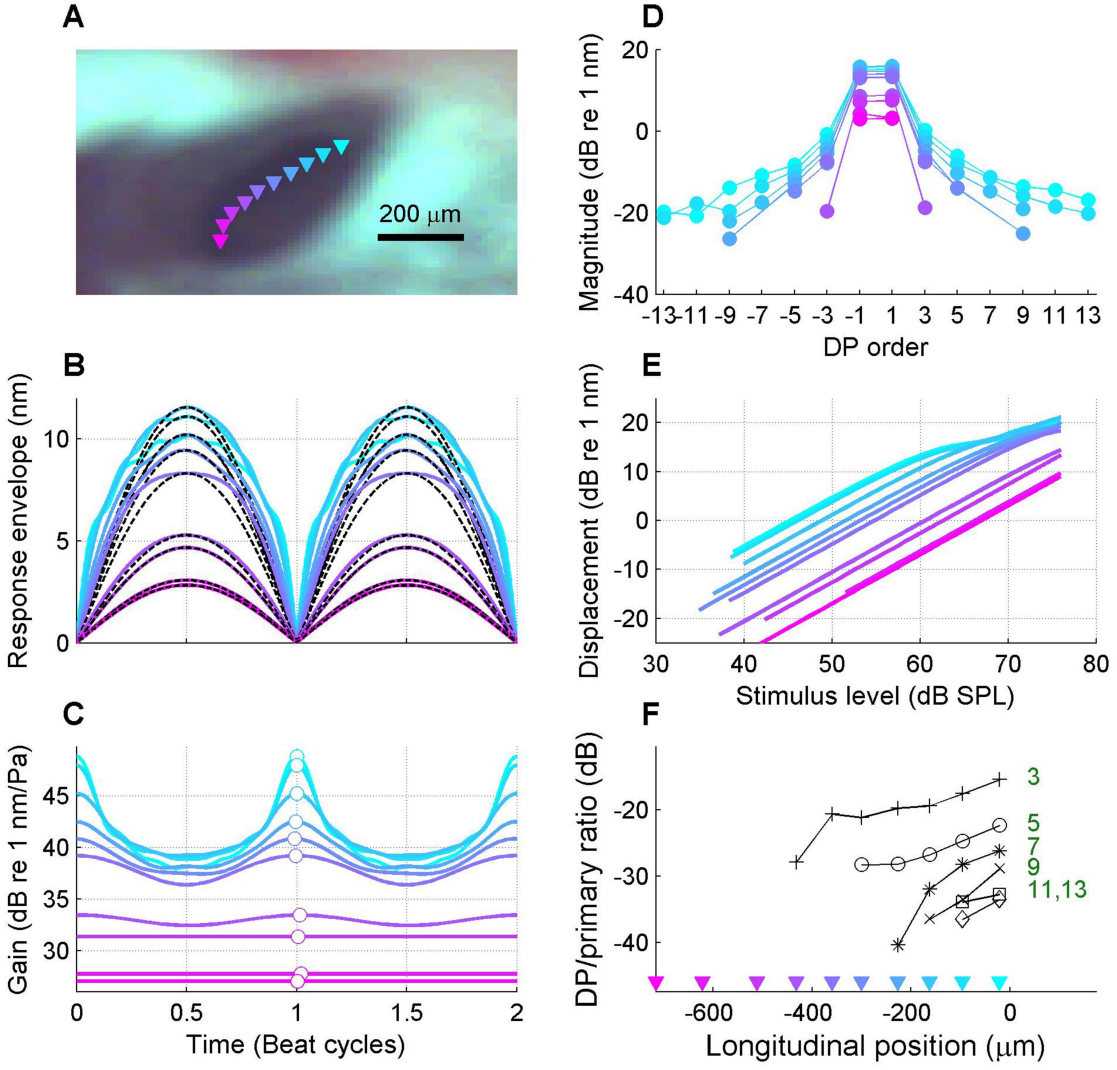
Gradual transition from linear to compressive responses along the length of the cochlea. **(A)** Video image of round window region with the OCT beam positions of the recording series superimposed (colored triangles). Best frequencies ranged from 23.8 down to 15.4 kHz. **(B)** Collection of BM response envelopes; stimulus frequency 15 kHz. Each curve shows data from a single location; the colors of the curves correspond to the beam positions indicated in panel **(A)**. **(C)** Corresponding time-varying gain curves. **(D)** Corresponding response spectra. **(E)** Corresponding I/O curves. **(F)** Magnitude ratio of distortion (sideband) components to primary components for each order of distortion, plotted as a function of longitudinal location from base to apex. This ratio serves as a metric for the local amount of compression (see text). The best place of the 15-kHz stimulus frequency is 0 μm by convention. Experiment RG211043.

In order to illustrate the transition from a linear response to a strongly compressive response in more spatial detail, Figure 5 shows data obtained from a series of BM locations spanning a longitudinal stretch of 695 μm.

The data in Figure 5 confirm that the nonlinear compression of the response to the stimulus is gradually accumulating from the most basal location (0 μm), where the response is linear to the peak location of the 15-kHz stimulus (~700 μm), where the response is strongly compressive. In addition to the response envelopes, instantaneous gain curves, spectra and I/O curves (Figures 5B–E), a spectral metric of compression is shown in Figure 5F, namely the magnitude ratio of the sidebands to the primary components. The idea is that the sidebands reflect the nonlinear distortion that comes with the compression; the stronger the compression, the stronger the sideband-to-primary ratio. This holds for sidebands of all orders (Van der Heijden, 2005). This correlation is not only expected from theoretical considerations; it is also clearly visible in the data when comparing the response spectra (Figure 5D) to the amount of compression shown by the gain curves (Figure 5C). The sideband-to-primary ratio quantifies this correlation and provides a simple metric whose spatial variation can be assessed in a straightforward way.

**Figure 6:**
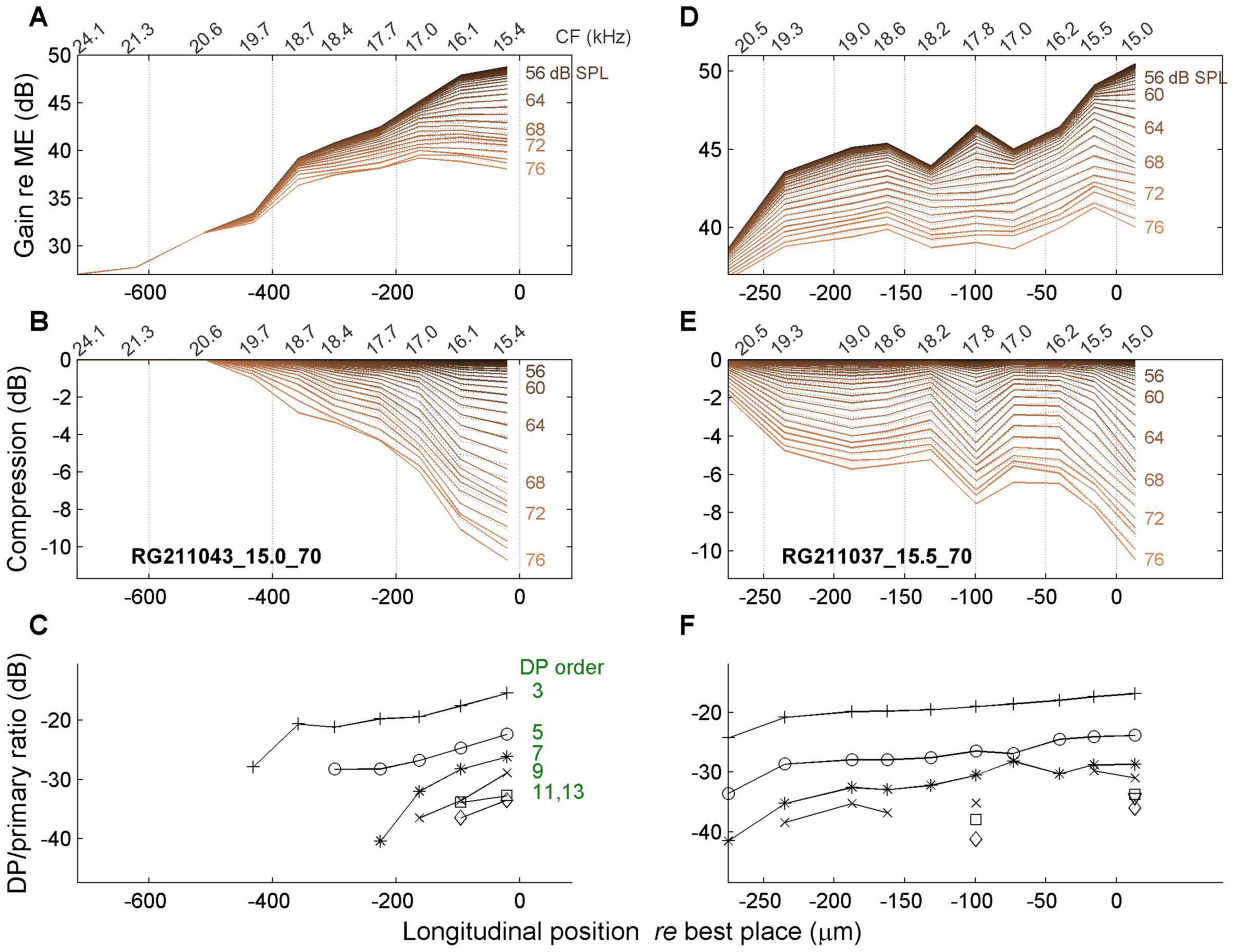
Spatial profiles of vibration magnitude, amount of compression and degree of distortion. In all panels, 0 μm corresponds to the best place of the stimulus frequency. **(A,D)** Magnitude of BM vibration, normalized to stapes motion, as a function of longitudinal location. Each curve represents a different SPL value (see text). **(B,E)** Corresponding spatial profiles of compression, i.e., the difference between gain at different SPL values and its maximum value occurring at the lowest SPL values. **(C,F)** Magnitude ratio of sidebands to primary components in the BM response as a function of longitudinal location. The order of the distortion is indicated in the graph. The two columns present data from different animals as indicated in panels **(B,E)**. Stimulus frequency, 15 kHz (left column); 15.5 kHz (right column).

**Figure 7:**
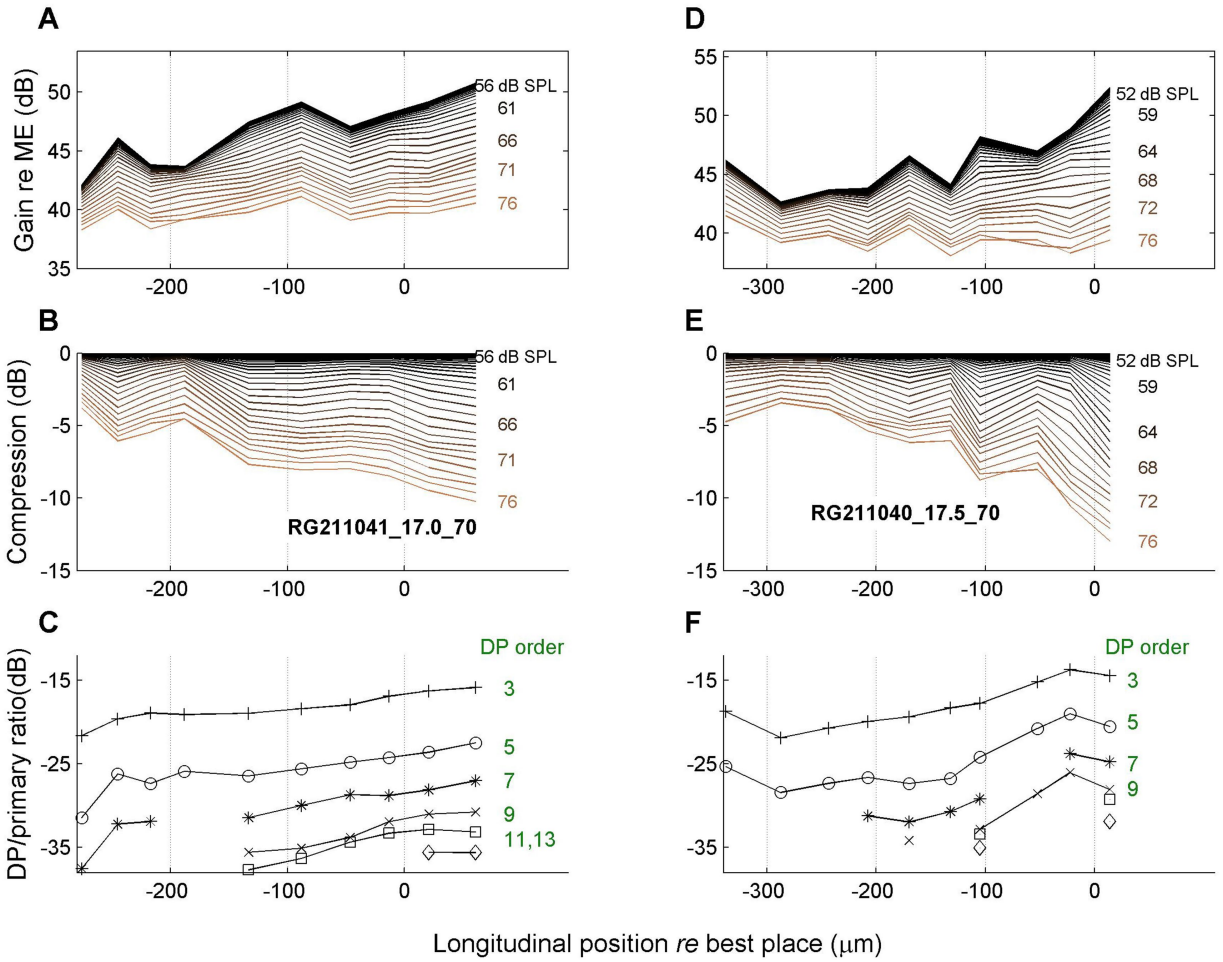
Spatial profiles as in Figure 7. Data of two more animals as indicated in panels **(B,E)**. Stimulus frequency, 17 kHz (left column); 17.5 kHz (right column).

A spatial depiction of the accumulation of compression can be realized by treating the different time points of the magnitude and gain curves as independent, “quasistatic” responses to single tones presented at different SPL values as they occur at consecutive time points. Specifically, the SPL values are extracted from the stimulus envelope (Figure 3A). The resulting spatial profiles are shown in Figures 6, 7 for four different cochleas. The upper rows (Figures 6A,D, 7A,D) show the magnitude of BM vibrations (normalized to stapes motion) as a function of longitudinal location. Each curve corresponds to a different SPL as indicated in the graphs.

The second row (Figures 6B,E, 7B,E) shows the amount of compression, i.e., the reduction of gain re the maximum value, as a function of longitudinal location. The spatial compression profiles in all four animals clearly show the systematic accumulation of compression along the direction of the traveling wave. The third row (Figures 6C,F, 7C,F) shows the corresponding sideband to primary ratio. This spectral metric of compression confirms the spatial buildup of nonlinear compression up to the peak of the traveling wave.

### 3.3 Nonlinear compression beyond the peak

The data of Figures 4–7 cover the growing flank of the traveling wave, i.e., the portion on the basal side of the peak. The observed growth of nonlinear compression from base to apex is consistent with the hypothesis of the spatial buildup of compression described in the Introduction, i.e., a framework in which the nonlinear compression at the peak is the result of a cascade of weakly compressive elements. On the other hand, the same data are also consistent with a very different mode of explanation in which compression reflects the operation of local nonlinear oscillators (e.g., Jülicher et al., 2001). The two modes of explanation, however, differ markedly in their prediction of the portion of the wave beyond the peak, at the apical flank. A cascade of nonlinear elements predicts that the amount of compression continues to accumulate beyond the peak. In contrast, a collection of uncoupled nonlinear oscillators predicts a gradual reduction of the amount of nonlinear compression beyond the peak. The latter, more symmetric pattern of compression is a direct consequence of the symmetric behavior of nonlinear oscillators: their response is maximally compressive at the best frequency and becomes more linear on both sides of the peak. To distinguish between the two scenarios, it is therefore necessary to study the spatial profile of compression at the apical flank of the peak of traveling wave.

**Figure 8:**
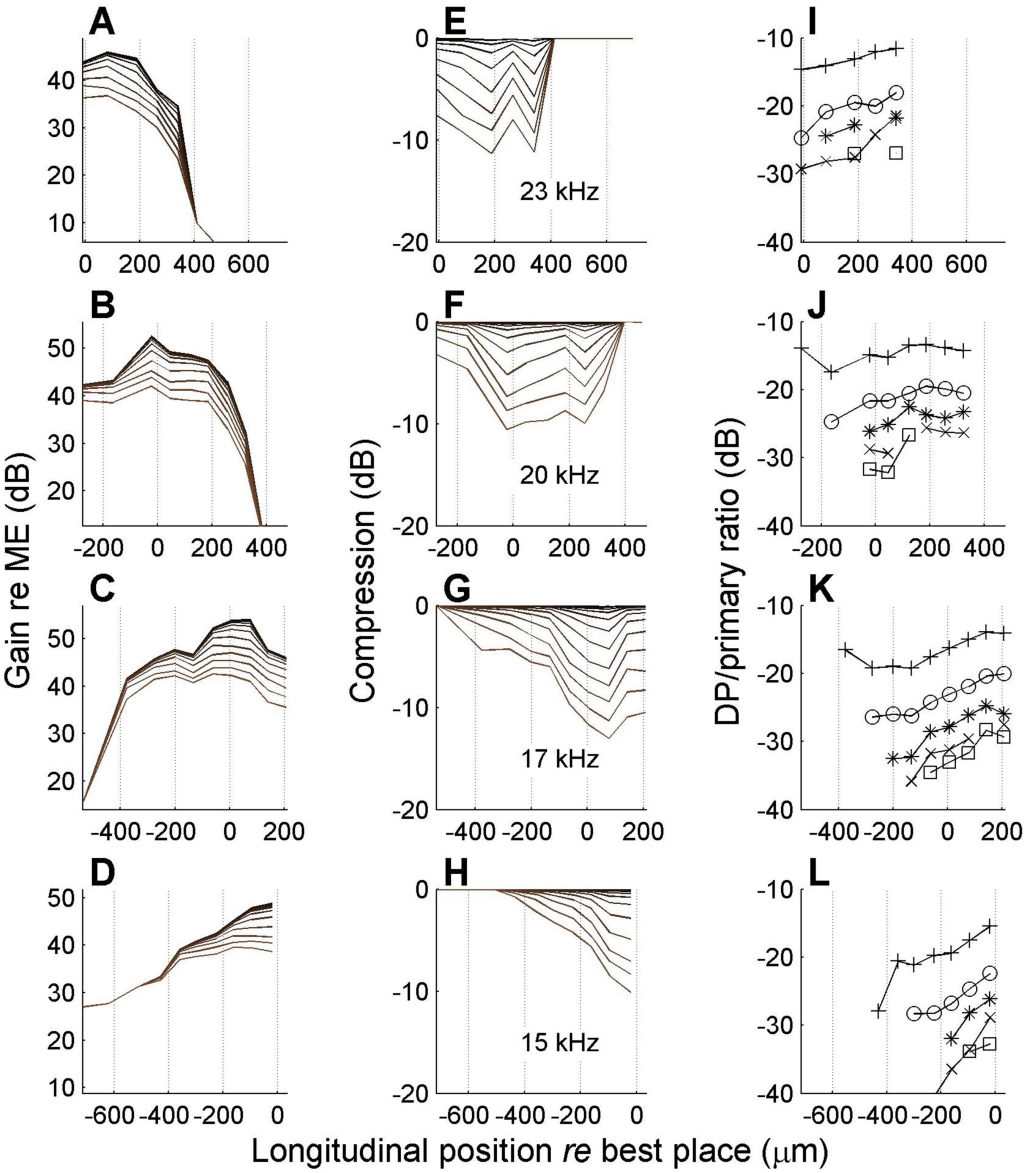
Spatial profiles of vibration magnitude, amount of compression and degree of distortion for different frequencies. Each row shows data obtained with a different stimulus frequency as indicated in the middle column. **(A–D)** Magnitude of BM vibration, normalized to stapes motion, as a function of longitudinal location. Different curves correspond to different SPLs as in Figure 6. **(E–H)** Corresponding spatial profiles of compression, i.e., the difference between gain at different SPL values and its maximum value occurring at the lowest SPL values. **(I,L)** Magnitude ratio of sidebands to primary components in the BM response as a function of longitudinal location. Animal RG211043. The physical range of longitudinal positions is identical in all panels, but, consistent with previous figures, 0 μm always corresponds to the best place of the stimulus frequency.

In one experiment we managed to record responses to many stimulus frequencies at the same series of longitudinal locations on the BM. This allowed us to study nonlinear compression in both flanks of the peak of the traveling wave. A representative set of results is shown in Figure 8.

The data in Figure 8 show that the spatial range over which compression occurred was ~800 μm, which is consistent with the longitudinal BM patterns of Ren (2002) and Fisher et al. (2012). With decreasing stimulus frequency (lower rows) the spatial peak and the compressive region shift toward the apex. The data in Figure 8 show a clear spatial asymmetry. The accumulation of nonlinear compression is gradual on the basal side of the peak (Figures 8F–H). Although the apical side of the peak also shows a return to linearity (zero compression in Figures 8E,F), this return to nonlinearity is much more abrupt. At first glance the abrupt disappearance of compression seems to contradict the hypothesis of a spatial buildup: in a cascade of compressive elements, the amount of compression can only accumulate. Any abrupt return to linearity (“plateau region,” e.g., Rhode, 2007) can be explained by an independent propagation mode (e.g., a pressure wave; Olson, 1998) that becomes dominant in the region beyond the peak where the slow traveling wave has decayed. But a gradual return to linearity would require a cascade of expansive elements that undo the compression that the wave has accumulated so far. This appears like a very unlikely scenario. Alternatively, such a gradual return to linearity may be evidence against the spatial buildup of compression and point toward a collection of uncoupled nonlinear oscillators.

The return to linearity in Figures 8E,F, however, may also be a spurious effect caused by the data analysis, which starts in the spectral domain (see Methods, 2.4). The gain and compression profiles are ultimately derived from the set of primary and sideband components that pass the Rayleigh test. Now the steep decay of the wave beyond its peak (Figures 8A,B) causes the sidebands to disappear in the noise floor just before the primaries themselves disappear. By construction, the disappearance of sidebands shows up in the analysis as a return to linearity, even though it is really more of a measurement problem. It is here that the spectral metric of compression, the sideband-to-primary ratio (Figures 8I–L) comes to the rescue. First of all it confirms that the apparent return to linearity in Figures 8E,F is indeed caused by a sudden disappearance of the sidebands, caused by their failure to pass the Rayleigh test. Furthermore, the sideband-to-primary confirms the spatial asymmetry of the compression. The systematic growth in the basal flank (Figures 8G,H) is not mirrored by a systematic decline in the apical flank (Figures 8I,J). In the apical flank the sideband-to-primary ratio keeps growing or saturates, eventually followed by a sudden disappearance of the sidebands.

### 3.4 Nonlinear compression inside the organ of Corti

**Figure 9:**
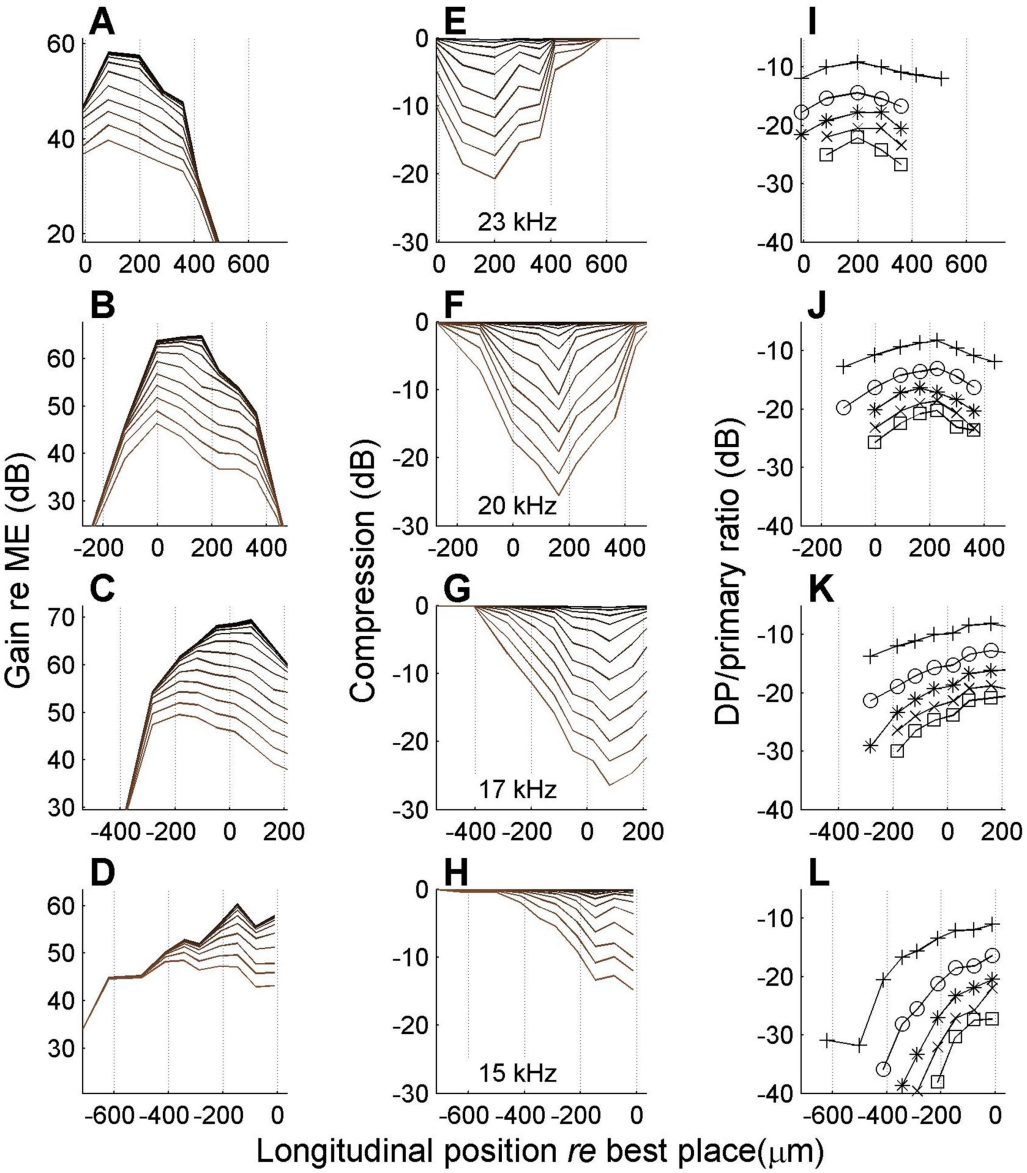
Spatial profiles of vibration magnitude, amount of compression and degree of distortion for different frequencies. Data obtained from the junction of Deiters’ cells and outer hair cells. Layout identical to Figure 8, same cochlea.

Figure 9 shows the same analysis as Figure 8, but now based on data recorded near the Deiters’ cell / outer hair cell junction (“hotspot” in Cooper et al., 2018) of the same animal (see Figure 2). There are several differences with the BM data of Figure 8. The amount of compression is larger in the hotspot than in the BM; this is consistent with data in the literature (e.g., Gao et al., 2014; Cooper et al., 2018; Strimbu et al., 2020). The decline of compression on the apical side is more gradual in the hotspot than in the BM (compare Figures 9E,F to Figures 8E,F), causing the spatial compression profiles to be more symmetric (Figure 9F). The more gradual decline of compression is also reflected in the profiles of the sideband-to-primary ratio (Figures 9I–L): instead of saturating and disappearing toward the apex, they show a mild reduction before disappearing (Figures 9K,L).

## 4 Discussion

Overall, the nonlinear longitudinal patterns of BM vibration confirm the spatial buildup of compression inferred from single-point recordings by Versteegh and van der Heijden (2013). Nonlinear growth at best place is indeed the culmination of a gradual buildup of compressive nonlinearity during wave propagation. In the base of the gerbil the nonlinear region starts ~0.5 mm basal to the best place. This coincides with the slow part of the traveling wave, and is consistent with a framework in which the slow wave propagation is exploited as a means to regulate sensitivity, most likely by controlling the amount of local damping based on local vibration amplitude.

As discussed more comprehensively in Cooper et al. (2018) and Van der Heijden and Vavakou (2022), an attractive feature of this framework is the clear functional role of traveling waves in the cochlea. In an alternative scheme, a parallel or “filterbank” design, each local filter or oscillator would need to be strongly compressive by itself over a huge dynamic range, and without abrupt saturation (hard clipping). This is a nontrivial engineering feat to achieve in a local circuit. In contrast, in a series or “cascade” design the workload of dynamic compression is neatly shared among a spatially distributed collection of regulators. The cumulative operation of a cascade ensures that each local link has only a modest role in the final result. Mathematically speaking, the effect of a cascade is characterized by exponential behavior (as a function of place), and small variations of the exponential coefficient (local damping) suffice to realize a considerable regulation of net gain. This is both simple and efficient, but the cascade design comes with a price, namely a potential interference across frequency bands in the form of suppression (physiology) and masking (psychophysics). This is the breakdown of the independence of sensitivity control across frequency bands mentioned in the Introduction: high-intensity, lower-frequency sounds interfere with the audibility of sound at (much) higher frequencies, an effect known in psychoacoustics as “upward spread of masking” (Wegel and Lane, 1924).

Beyond the peak of the traveling wave, at its apical flank, the BM vibrations showed a continued accumulation of nonlinear compression. In our view this spatial asymmetry is the strongest indication of the inherent directionality of the dynamic range compression in the cochlea. It is clearly inconsistent with the view of the cochlea as a set of uncoupled oscillators. In fact it is not obvious that adapting such a “filterbank” framework by incorporating a coupling between adjacent oscillators (Duifhuis et al., 1986) would correctly reproduce the unidirectional character of compression observed in our data. On the other hand, the unidirectionality of the nonlinear compression as reported in this study does not necessarily imply the unidirectionality of the compressive mechanism itself. Two broad classes of models are both compatible with our findings. In the first class, the variable forward gain stems from a local regulation of damping; either negative (Neely and Kim, 1983; de Boer 1983) or positive (van der Heijden and Vavakou, 2022). Damping itself is not directional: it equally affects waves forward and reverse traveling waves. In this class of models the unidirectional buildup stems from the unidirectional context in which damping does its job, namely, the traveling wave. In the second class of models, directionality is an inherent property of the compressive mechanism; it is built into the system in the sense that each segment passes its excitation only to its apical neighbor. Such a nonreciprocal transfer of excitation is not a waveguide in the classical sense. It is more properly described as a cascade. Cochlear models of this inherently unidirectional type include Kim et al. (1973), Lyon (1982), Geisler and Sang (1995), and experimental support for unidirectional coupling includes Ren (2004), and Ren et al. (2014). The findings of the current study, however, cannot be used to decide between the two classes of models.

The nonlinear compression profiles inside the organ of Corti (Figure 9) were also spatially asymmetric, but could not be characterized by a purely unidirectional accumulation of nonlinearity. This difference in the longitudinal compression profiles between BM and structures inside the organ of Corti is not easy to interpret, but it should be noted that the organ of Corti vibrations have a number of other features that present qualitative differences with the BM: compression over a much wider frequency range down to frequencies many octaves below the best frequency; the higher “threshold SPL” needed for the responses to become compressive over a wide frequency range; large phase leads *re* the BM in the low-frequency tail; the suppressibility of low-frequency responses by higher frequency tones (Gao et al., 2014; Cooper et al., 2018; Dewey et al., 2019). Importantly, the wideband compression, even though it disappears post mortem, is physiologically much more robust than the tuned, BM-like compression (Strimbu et al., 2020). These qualitative differences have led to the proposal that the wideband compression observed in the organ of Corti has a different origin than the “classical” narrowband BM nonlinearity and is more locally generated (Cooper et al., 2018; Dewey et al., 2019). The data in Figure 9 are consistent with such a local contribution to the nonlinearity. In this interpretation, the observed pattern of compression is a superposition of a propagating, tuned, nonlinearity and a local, untuned nonlinearity. The decay of the traveling wave beyond its peak causes its amplitude to fall below the threshold for the wideband compression, and this explains the slight reduction of compression beyond the peak, where only the propagating, tuned compression persists.

Our data are restricted to the high-frequency, basal region of the cochlea, and one may question whether our conclusions equally apply to other cochlear regions. It is sometimes argued that the mechanics in the apex is qualitatively different (e.g., Burwood et al., 2022; for a recent discussion of this “apical exceptionalism,” see Recio-Spinoso et al., 2023). Although we have only presented basal data in this study, we believe that the spatial buildup of compression, and in particular the crucial role in cochlear nonlinearity played by the traveling wave, reflect generic cochlear mechanisms. The nonlinear phenomena naturally explained by the spatial buildup (Versteegh and van der Heijden, 2013) have been found throughout the cochlea. The systematic decrease of I/O slopes with frequency has been reported in the 2–3-kHz region (middle turn) of the gerbil BM (Meenderink et al., 2022) and in the 400-Hz region of the chinchilla tectorial membrane (Rhode and Cooper, 1996). Systematic decrease of the rate of suppression with increasing suppressor frequency has been found for the vibrations in the chinchilla apex for probe frequencies ranging from 200 to 800 Hz (Cooper and Rhode, 1996). The systematic effect of suppressor frequency on the rate of suppression in auditory nerve fibers has been found over a large range of characteristic frequencies, e.g., 17.8 kHz (Abbas and Sachs, 1976); 540 Hz (Delgutte, 1990). Its psychoacoustic counterpart, the effect of masker frequency on the growth of masking has been reported for a wide range of signal frequencies, as low as 600 Hz (Wegel and Lane, 1924) and 250 Hz (Glasberg and Moore, 1994). Many of these data suggest that the degree of nonlinearity is lower in the apex than in the base, but all of the data share the consistent feature that the slopes of I/O curves and the rate of suppression decrease with increasing frequency. Thus there appears to be no compelling reason to invoke qualitative differences in the mechanisms of compression between base and apex; they seem to share the same buildup of compression along the traveling wave.

## Data Availability

The raw data supporting the conclusions of this article will be made available by the authors, without undue reservation. Data underlying the figures in this article can be found at https://doi.org/10.6084/m9.figshare.28079705.
